# [1-(3-Chloro­phen­yl)-1*H*-1,2,3-triazol-4-yl]methanol hemihydrate

**DOI:** 10.1107/S1600536811041560

**Published:** 2011-10-12

**Authors:** Nübia Boechat, Maria de Lourdes G. Ferreira, Monica M. Bastos, James L. Wardell, Solange M. S. V. Wardell, Edward R. T. Tiekink

**Affiliations:** a Fundação Oswaldo Cruz, Instituto de Tecnologia em Fármacos, Departamento de Sintese Organica, Manguinhos, 21041-250 Rio de Janeiro, RJ, Brazil; b Centro de Desenvolvimento Tecnológico em Saúde (CDTS), Fundação Oswaldo Cruz (FIOCRUZ), Casa Amarela, Campus de Manguinhos, Av. Brasil 4365, 21040-900 Rio de Janeiro, RJ, Brazil; c CHEMSOL, 1 Harcourt Road, Aberdeen AB15 5NY, Scotland; d Department of Chemistry, University of Malaya, 50603 Kuala Lumpur, Malaysia

## Abstract

The asymmetric unit of the title hydrate, C_9_H_8_ClN_3_O·0.5H_2_O, comprises two independent 1,2,3-triazole mol­ecules and a water mol­ecule of crystallization. The dihedral angles between the six- and five-membered rings in the 1,2,3-triazole mol­ecules are 12.71 (19) and 17.3 (2)°. The most significant different between them is found in the relative orientations of the terminal CH_2_OH groups with one being close to perpendicular to the five-membered ring [N—C—C—O torsion angle = 82.2 (5)°], while in the other mol­ecule, a notable deviation from a perpendicular disposition is found [torsion angle = −60.3 (5)°]. Supra­molecular chains feature in the crystal packing sustained by O—H⋯(O,N) inter­actions along the *a*-axis direction. The chains are connected *via* C—H⋯N inter­actions and the resultant layers stack along the *b* axis.

## Related literature

For background to the synthesis, biological activity and structures of 1,2,3-triazole derivatives, see: Boechat *et al.* (2010 ▶, 2011 ▶); Costa *et al.* (2006*a* ▶,*b* ▶); Ferreira *et al.* (2007 ▶); Jordão *et al.* (2009 ▶). For the synthesis, see: Boechat *et al.* (2011 ▶). For additional geometric analysis, see: Spek (2009 ▶).
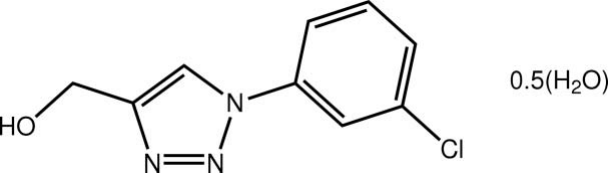


## Experimental

### 

#### Crystal data

C_9_H_8_ClN_3_O·0.5H_2_O
*M* *_r_* = 218.64
Triclinic, 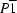

*a* = 6.0078 (4) Å
*b* = 7.4897 (4) Å
*c* = 22.3145 (15) Å
α = 88.818 (4)°
β = 89.901 (2)°
γ = 80.493 (4)°
*V* = 990.07 (11) Å^3^
*Z* = 4
Mo *K*α radiation
μ = 0.36 mm^−1^
*T* = 120 K
0.18 × 0.18 × 0.02 mm

#### Data collection

Bruker–Nonius APEX II CCD camera on κ-goniostat diffractometer
Absorption correction: multi-scan (*SADABS*; Sheldrick, 2007 ▶) *T*_min_ = 0.843, *T*_max_ = 1.000
10830 measured reflections
3909 independent reflections
2948 reflections with *I* > 2σ(*I*)
*R*_int_ = 0.038

#### Refinement

*R*[*F* ^2^ > 2σ(*F* ^2^)] = 0.064
*wR*(*F* ^2^) = 0.163
*S* = 1.00
3909 reflections
274 parameters
5 restraints
H atoms treated by a mixture of independent and constrained refinement
Δρ_max_ = 0.41 e Å^−3^
Δρ_min_ = −0.34 e Å^−3^

### 

Data collection: *COLLECT* (Hooft, 1998 ▶); cell refinement: *DENZO* (Otwinowski & Minor, 1997 ▶) and *COLLECT*; data reduction: *DENZO* and *COLLECT*; program(s) used to solve structure: *SHELXS97* (Sheldrick, 2008 ▶); program(s) used to refine structure: *SHELXL97* (Sheldrick, 2008 ▶); molecular graphics: *ORTEP-3* (Farrugia, 1997 ▶), *QMol* (Gans & Shalloway, 2001 ▶) and *DIAMOND* (Brandenburg, 2006 ▶); software used to prepare material for publication: *publCIF* (Westrip, 2010 ▶).

## Supplementary Material

Crystal structure: contains datablock(s) global, I. DOI: 10.1107/S1600536811041560/hb6439sup1.cif

Structure factors: contains datablock(s) I. DOI: 10.1107/S1600536811041560/hb6439Isup2.hkl

Supplementary material file. DOI: 10.1107/S1600536811041560/hb6439Isup3.cml

Additional supplementary materials:  crystallographic information; 3D view; checkCIF report

## Figures and Tables

**Table 1:** Hydrogen-bond geometry (Å, °)

*D*—H⋯*A*	*D*—H	H⋯*A*	*D*⋯*A*	*D*—H⋯*A*
O1—H1o⋯O2^i^	0.84 (4)	1.82 (4)	2.651 (5)	170 (5)
O2—H2o⋯O1w	0.84 (6)	1.80 (5)	2.641 (5)	174 (7)
O1w—H1w⋯N3	0.84 (4)	2.00 (4)	2.837 (5)	172 (4)
O1w—H2w⋯O1^ii^	0.84 (4)	1.95 (5)	2.663 (5)	142 (4)
C16—H16⋯O1w^iii^	0.95	2.45	3.383 (5)	166
C7—H7⋯N6^iv^	0.95	2.28	3.197 (5)	161

Symmetry codes: (i) 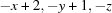; (ii) 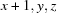; (iii) 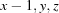; (iv) 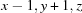.

**Fig. 1.**
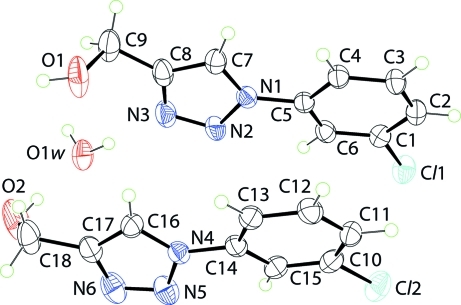
The molecular structures of the components comprising the asymmetric unit in (I) showing displacement ellipsoids at the 50% probability level.

**Fig. 2.**
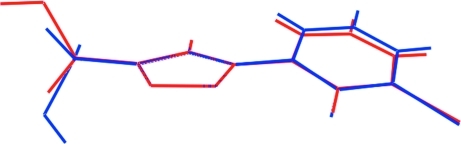
An overlay diagram of the two independent molecules in (I). The red and blue images illustrate the N1- and N3-containing molecules, respectively.

**Fig. 3.**
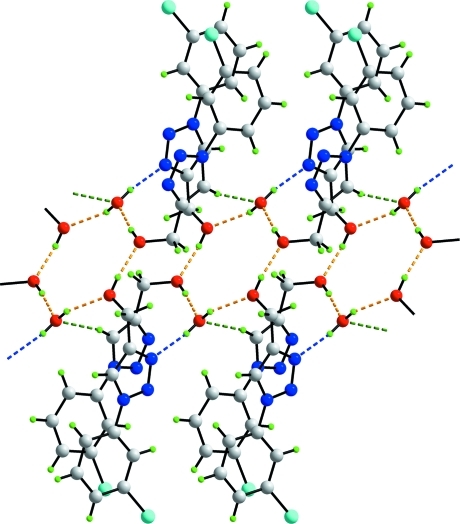
A view of the supramolecular chain aligned along the a axis in (I) mediated by O—H···O (red dashed lines), O—H···N (blue) hydrogen bonds and C—H···O interactions (green).

**Fig. 4.**
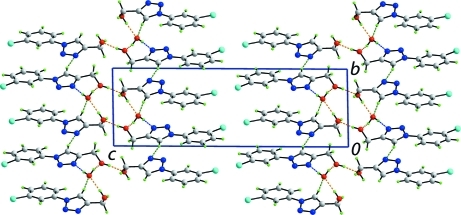
A view in projection down the a axis of the unit-cell contents in (I) showing the stacking of layers along the b axis. The O—H···O, O—H···N and C—H···O interactions are shown as orange, blue and green dashed lines, respectively.

## supplementary crystallographic information

### Comment

Boechat and colleagues have been interested in the synthesis, biological activities and structures of 1,2,3-triazole derivatives for some time (Boechat *et al.*, 2010, 2011; Costa *et al.*, 2006*a*, 2006*b*; Ferreira *et al.*, 2007, Jordão *et al.*, 2009). Recently, they reported the synthesis and anti-mycobacterial activities of a number of 4-*R*-1-(*X*-phenyl)-triazole derivatives (Boechat *et al.*, 2011). The structure of one of the compounds investigated in that study, *i.e*. the title compound, (I), is now reported.

Two independent molecules of a 1,2,3-triazole derivative and a water molecule of solvation comprise the asymmetric unit of (I), Fig. 1. Geometrically, the two organic molecules are similar to each other with r.m.s. deviations for bond distances and angles being 0.0092 Å and 0.757°, respectively (Spek, 2009). From the overlay diagram, Fig. 2, it is evident that the independent molecules approximate mirror images. However, small twists between the five- and six-membered rings differ with the dihedral angles between their least-squares being 12.71 (19) and 17.3 (2)°, respectively, for the N1- and N4-containing molecules. More notable are the relative orientations of the terminal CH_2_OH groups as seen in the values of the N3—C8—C9—O1 and N6—C17—C18—O2 torsion angles of 82.2 (5) and -60.3 (5)°, respectively.

The presence of a supramolecular chain along the *a* axis is the most prominent feature of the crystal packing, Fig. 3. These are mediated by O—H···O and O—H···N hydrogen bonds with additional stability afforded by C—H···O interactions, Table 1. Chains are connected into layers *via* C—H···N interactions, Table 1, and these stack along the *b* axis. The closest interactions between layers are of the type Cl···Cl, *i.e*. Cl1···Cl2^i^ = 3.4117 (15) Å for *i*: 2 - *x*, 1 - *y*, 1 - *z*.

### Experimental

The compound, obtained as published (Boechat *et al.*, 2011), was recrystallized from EtOH as a hemihydrate.

### Refinement

The C-bound H atoms were geometrically placed (C—H = 0.95–0.99 Å) and refined as riding with *U_iso_*(H) = 1.2*U_eq_*(C). The O—H H atoms were located from a difference map and refined with O—H = 0.84±0.01 Å, and with *U_iso_*(H) = 1.5*U_eq_*(O).

### Crystal data

**Table:**

C_9_H_8_ClN_3_O·0.5H_2_O	*Z* = 4
*M**_r_* = 218.64	*F*(000) = 452
Triclinic, *P*1	*D*_x_ = 1.467 Mg m^−^^3^
Hall symbol: -P 1	Mo *K*α radiation, λ = 0.71073 Å
*a* = 6.0078 (4) Å	Cell parameters from 19812 reflections
*b* = 7.4897 (4) Å	θ = 2.9–27.5°
*c* = 22.3145 (15) Å	µ = 0.36 mm^−^^1^
α = 88.818 (4)°	*T* = 120 K
β = 89.901 (2)°	Plate, colourless
γ = 80.493 (4)°	0.18 × 0.18 × 0.02 mm
*V* = 990.07 (11) Å^3^	

### Data collection

**Table:**

Bruker–Nonius APEX II CCD camera on κ-goniostat diffractometer	3909 independent reflections
Radiation source: Bruker-Nonius FR591 rotating anode	2948 reflections with *I* > 2σ(*I*)
10cm confocal mirrors	*R*_int_ = 0.038
Detector resolution: 9.091 pixels mm^-1^	θ_max_ = 26.5°, θ_min_ = 2.9°
φ and ω scans	*h* = −7→7
Absorption correction: multi-scan (*SADABS*; Sheldrick, 2007)	*k* = −9→9
*T*_min_ = 0.843, *T*_max_ = 1.000	*l* = −28→28
10830 measured reflections	

### Refinement

**Table:**

Refinement on *F*^2^	Primary atom site location: structure-invariant direct methods
Least-squares matrix: full	Secondary atom site location: difference Fourier map
*R*[*F*^2^ > 2σ(*F*^2^)] = 0.064	Hydrogen site location: inferred from neighbouring sites
*wR*(*F*^2^) = 0.163	H atoms treated by a mixture of independent and constrained refinement
*S* = 1.00	*w* = 1/[σ^2^(*F*_o_^2^) + (0.0446*P*)^2^ + 2.5602*P*] where *P* = (*F*_o_^2^ + 2*F*_c_^2^)/3
3909 reflections	(Δ/σ)_max_ = 0.012
274 parameters	Δρ_max_ = 0.41 e Å^−^^3^
5 restraints	Δρ_min_ = −0.34 e Å^−^^3^

### Special details

**Table:**

Geometry. All s.u.'s (except the s.u. in the dihedral angle between two l.s. planes) are estimated using the full covariance matrix. The cell s.u.'s are taken into account individually in the estimation of s.u.'s in distances, angles and torsion angles; correlations between s.u.'s in cell parameters are only used when they are defined by crystal symmetry. An approximate (isotropic) treatment of cell s.u.'s is used for estimating s.u.'s involving l.s. planes.
Refinement. Refinement of *F*^2^ against ALL reflections. The weighted *R*-factor *wR* and goodness of fit *S* are based on *F*^2^, conventional *R*-factors *R* are based on *F*, with *F* set to zero for negative *F*^2^. The threshold expression of *F*^2^ > 2σ(*F*^2^) is used only for calculating *R*-factors(gt) *etc*. and is not relevant to the choice of reflections for refinement. *R*-factors based on *F*^2^ are statistically about twice as large as those based on *F*, and *R*- factors based on ALL data will be even larger.

### Fractional atomic coordinates and isotropic or equivalent isotropic displacement parameters (Å^2^)

**Table:**

	*x*	*y*	*z*	*U*_iso_*/*U*_eq_	
Cl1	0.85344 (17)	0.75800 (15)	0.46687 (4)	0.0449 (3)	
O1	0.5639 (6)	0.7737 (5)	0.05307 (13)	0.0595 (9)	
H1O	0.629 (9)	0.747 (7)	0.0204 (13)	0.089*	
N1	0.6411 (5)	0.8503 (4)	0.24523 (13)	0.0297 (6)	
N2	0.8433 (5)	0.7528 (4)	0.23011 (14)	0.0386 (7)	
N3	0.8614 (6)	0.7674 (4)	0.17193 (14)	0.0424 (8)	
C1	0.6580 (6)	0.8250 (5)	0.41004 (15)	0.0304 (7)	
C2	0.4384 (6)	0.8954 (5)	0.42488 (16)	0.0343 (8)	
H2	0.3932	0.9055	0.4657	0.041*	
C3	0.2852 (6)	0.9509 (5)	0.37926 (15)	0.0331 (8)	
H3	0.1338	1.0005	0.3889	0.040*	
C4	0.3494 (6)	0.9352 (5)	0.31987 (16)	0.0317 (8)	
H4	0.2426	0.9724	0.2889	0.038*	
C5	0.5708 (6)	0.8647 (4)	0.30588 (15)	0.0280 (7)	
C6	0.7280 (6)	0.8092 (5)	0.35095 (15)	0.0314 (8)	
H6	0.8801	0.7614	0.3414	0.038*	
C7	0.5315 (6)	0.9254 (5)	0.19577 (15)	0.0322 (8)	
H7	0.3870	0.9992	0.1940	0.039*	
C8	0.6716 (7)	0.8731 (5)	0.14894 (16)	0.0381 (9)	
C9	0.6412 (8)	0.9160 (6)	0.08392 (17)	0.0484 (11)	
H9A	0.5309	1.0289	0.0784	0.058*	
H9B	0.7867	0.9368	0.0665	0.058*	
Cl2	0.7137 (2)	0.31120 (15)	0.43211 (4)	0.0519 (3)	
O2	1.1922 (7)	0.3320 (5)	0.04279 (18)	0.0843 (14)	
H2O	1.219 (12)	0.422 (6)	0.062 (3)	0.126*	
N4	0.7744 (5)	0.2978 (4)	0.20400 (13)	0.0306 (6)	
N5	0.9702 (5)	0.1798 (5)	0.21008 (15)	0.0433 (8)	
N6	1.0590 (6)	0.1604 (5)	0.15647 (16)	0.0485 (9)	
C10	0.5956 (6)	0.3531 (5)	0.36144 (16)	0.0348 (8)	
C11	0.3706 (6)	0.4305 (5)	0.35577 (16)	0.0359 (8)	
H11	0.2793	0.4587	0.3902	0.043*	
C12	0.2826 (6)	0.4657 (5)	0.29895 (17)	0.0389 (9)	
H12	0.1293	0.5210	0.2943	0.047*	
C13	0.4137 (6)	0.4217 (5)	0.24830 (16)	0.0318 (8)	
H13	0.3506	0.4445	0.2093	0.038*	
C14	0.6367 (6)	0.3444 (4)	0.25557 (16)	0.0309 (8)	
C15	0.7315 (6)	0.3090 (5)	0.31195 (16)	0.0323 (8)	
H15	0.8855	0.2558	0.3166	0.039*	
C16	0.7421 (6)	0.3525 (5)	0.14617 (16)	0.0350 (8)	
H16	0.6188	0.4348	0.1298	0.042*	
C17	0.9245 (7)	0.2643 (5)	0.11626 (18)	0.0395 (9)	
C18	0.9826 (8)	0.2697 (6)	0.05057 (19)	0.0538 (12)	
H18A	0.9928	0.1471	0.0339	0.065*	
H18B	0.8623	0.3516	0.0287	0.065*	
O1W	1.2489 (5)	0.6147 (4)	0.10634 (13)	0.0491 (7)	
H1W	1.143 (6)	0.661 (6)	0.1286 (19)	0.074*	
H2W	1.293 (8)	0.698 (5)	0.086 (2)	0.074*	

### Atomic displacement parameters (Å^2^)

**Table:**

	*U*^11^	*U*^22^	*U*^33^	*U*^12^	*U*^13^	*U*^23^
Cl1	0.0387 (5)	0.0620 (7)	0.0332 (5)	−0.0063 (4)	−0.0052 (4)	0.0013 (4)
O1	0.081 (2)	0.077 (2)	0.0334 (15)	−0.0501 (19)	0.0214 (15)	−0.0252 (15)
N1	0.0308 (16)	0.0279 (15)	0.0314 (15)	−0.0076 (12)	0.0057 (12)	−0.0036 (12)
N2	0.0380 (18)	0.0384 (18)	0.0380 (17)	−0.0016 (14)	0.0155 (14)	−0.0037 (13)
N3	0.049 (2)	0.0433 (19)	0.0375 (18)	−0.0135 (15)	0.0174 (16)	−0.0073 (14)
C1	0.0303 (18)	0.0333 (19)	0.0288 (17)	−0.0085 (14)	−0.0026 (14)	−0.0010 (14)
C2	0.037 (2)	0.039 (2)	0.0283 (18)	−0.0112 (16)	0.0055 (16)	−0.0051 (15)
C3	0.0289 (18)	0.039 (2)	0.0310 (18)	−0.0040 (15)	0.0073 (15)	−0.0030 (15)
C4	0.0339 (19)	0.0316 (19)	0.0298 (18)	−0.0061 (14)	0.0050 (15)	−0.0024 (14)
C5	0.0308 (18)	0.0259 (17)	0.0283 (17)	−0.0074 (13)	0.0036 (14)	−0.0025 (13)
C6	0.0280 (18)	0.0317 (19)	0.0348 (19)	−0.0056 (14)	0.0070 (15)	−0.0043 (14)
C7	0.0345 (19)	0.0354 (19)	0.0290 (18)	−0.0120 (15)	0.0024 (15)	−0.0039 (14)
C8	0.048 (2)	0.038 (2)	0.0327 (19)	−0.0193 (17)	0.0104 (17)	−0.0109 (16)
C9	0.070 (3)	0.051 (3)	0.032 (2)	−0.032 (2)	0.014 (2)	−0.0089 (18)
Cl2	0.0633 (7)	0.0630 (7)	0.0319 (5)	−0.0187 (5)	−0.0054 (5)	0.0050 (4)
O2	0.093 (3)	0.094 (3)	0.085 (3)	−0.067 (2)	0.063 (2)	−0.049 (2)
N4	0.0293 (15)	0.0294 (15)	0.0333 (16)	−0.0048 (12)	0.0040 (12)	−0.0042 (12)
N5	0.0298 (17)	0.050 (2)	0.047 (2)	0.0020 (14)	0.0020 (15)	−0.0105 (15)
N6	0.0359 (19)	0.057 (2)	0.053 (2)	−0.0086 (16)	0.0100 (17)	−0.0177 (17)
C10	0.043 (2)	0.034 (2)	0.0289 (18)	−0.0136 (16)	−0.0044 (16)	0.0042 (14)
C11	0.041 (2)	0.036 (2)	0.0318 (19)	−0.0101 (16)	0.0087 (16)	−0.0011 (15)
C12	0.034 (2)	0.036 (2)	0.045 (2)	−0.0018 (16)	0.0087 (17)	−0.0016 (16)
C13	0.0333 (19)	0.0312 (19)	0.0316 (18)	−0.0075 (14)	−0.0006 (15)	−0.0011 (14)
C14	0.0329 (19)	0.0259 (18)	0.0355 (19)	−0.0087 (14)	0.0080 (15)	−0.0039 (14)
C15	0.0308 (19)	0.0333 (19)	0.0340 (19)	−0.0086 (14)	−0.0009 (15)	0.0027 (14)
C16	0.041 (2)	0.034 (2)	0.0316 (19)	−0.0113 (16)	0.0063 (16)	−0.0002 (15)
C17	0.044 (2)	0.038 (2)	0.042 (2)	−0.0190 (17)	0.0149 (18)	−0.0105 (17)
C18	0.061 (3)	0.063 (3)	0.046 (2)	−0.033 (2)	0.026 (2)	−0.019 (2)
O1W	0.0453 (17)	0.065 (2)	0.0381 (16)	−0.0109 (14)	0.0155 (13)	−0.0064 (14)

### Geometric parameters (Å, °)

**Table:**

Cl1—C1	1.739 (4)	O2—C18	1.423 (5)
O1—C9	1.422 (5)	O2—H2O	0.840 (10)
O1—H1O	0.839 (10)	N4—C16	1.350 (4)
N1—C7	1.350 (4)	N4—N5	1.355 (4)
N1—N2	1.356 (4)	N4—C14	1.431 (4)
N1—C5	1.418 (4)	N5—N6	1.310 (5)
N2—N3	1.307 (4)	N6—C17	1.353 (5)
N3—C8	1.370 (5)	C10—C11	1.385 (5)
C1—C2	1.380 (5)	C10—C15	1.385 (5)
C1—C6	1.385 (5)	C11—C12	1.378 (5)
C2—C3	1.384 (5)	C11—H11	0.9500
C2—H2	0.9500	C12—C13	1.390 (5)
C3—C4	1.382 (5)	C12—H12	0.9500
C3—H3	0.9500	C13—C14	1.377 (5)
C4—C5	1.385 (5)	C13—H13	0.9500
C4—H4	0.9500	C14—C15	1.384 (5)
C5—C6	1.390 (5)	C15—H15	0.9500
C6—H6	0.9500	C16—C17	1.364 (5)
C7—C8	1.363 (5)	C16—H16	0.9500
C7—H7	0.9500	C17—C18	1.507 (5)
C8—C9	1.485 (5)	C18—H18A	0.9900
C9—H9A	0.9900	C18—H18B	0.9900
C9—H9B	0.9900	O1W—H1W	0.840 (10)
Cl2—C10	1.731 (4)	O1W—H2W	0.838 (10)
			
C9—O1—H1O	115 (4)	C16—N4—N5	110.3 (3)
C7—N1—N2	110.3 (3)	C16—N4—C14	130.2 (3)
C7—N1—C5	128.6 (3)	N5—N4—C14	119.5 (3)
N2—N1—C5	121.1 (3)	N6—N5—N4	106.6 (3)
N3—N2—N1	106.9 (3)	N5—N6—C17	109.9 (3)
N2—N3—C8	109.7 (3)	C11—C10—C15	121.9 (3)
C2—C1—C6	121.7 (3)	C11—C10—Cl2	119.7 (3)
C2—C1—Cl1	119.3 (3)	C15—C10—Cl2	118.5 (3)
C6—C1—Cl1	119.0 (3)	C12—C11—C10	118.3 (3)
C1—C2—C3	118.8 (3)	C12—C11—H11	120.8
C1—C2—H2	120.6	C10—C11—H11	120.8
C3—C2—H2	120.6	C11—C12—C13	121.3 (4)
C4—C3—C2	120.9 (3)	C11—C12—H12	119.4
C4—C3—H3	119.6	C13—C12—H12	119.4
C2—C3—H3	119.6	C14—C13—C12	118.9 (3)
C3—C4—C5	119.5 (3)	C14—C13—H13	120.6
C3—C4—H4	120.3	C12—C13—H13	120.6
C5—C4—H4	120.3	C13—C14—C15	121.4 (3)
C6—C5—C4	120.7 (3)	C13—C14—N4	119.7 (3)
C6—C5—N1	119.0 (3)	C15—C14—N4	118.8 (3)
C4—C5—N1	120.4 (3)	C14—C15—C10	118.2 (3)
C5—C6—C1	118.5 (3)	C14—C15—H15	120.9
C5—C6—H6	120.7	C10—C15—H15	120.9
C1—C6—H6	120.7	N4—C16—C17	105.2 (3)
N1—C7—C8	105.6 (3)	N4—C16—H16	127.4
N1—C7—H7	127.2	C17—C16—H16	127.4
C8—C7—H7	127.2	N6—C17—C16	108.0 (3)
C7—C8—N3	107.5 (3)	N6—C17—C18	122.0 (4)
C7—C8—C9	129.8 (4)	C16—C17—C18	129.9 (4)
N3—C8—C9	122.7 (3)	O2—C18—C17	109.9 (4)
O1—C9—C8	111.6 (3)	O2—C18—H18A	109.7
O1—C9—H9A	109.3	C17—C18—H18A	109.7
C8—C9—H9A	109.3	O2—C18—H18B	109.7
O1—C9—H9B	109.3	C17—C18—H18B	109.7
C8—C9—H9B	109.3	H18A—C18—H18B	108.2
H9A—C9—H9B	108.0	H1W—O1W—H2W	109 (4)
C18—O2—H2O	120 (5)		
			
C7—N1—N2—N3	0.5 (4)	C16—N4—N5—N6	−0.2 (4)
C5—N1—N2—N3	−179.0 (3)	C14—N4—N5—N6	179.2 (3)
N1—N2—N3—C8	−0.3 (4)	N4—N5—N6—C17	0.3 (4)
C6—C1—C2—C3	0.1 (5)	C15—C10—C11—C12	−0.7 (5)
Cl1—C1—C2—C3	179.0 (3)	Cl2—C10—C11—C12	178.7 (3)
C1—C2—C3—C4	0.5 (5)	C10—C11—C12—C13	1.3 (5)
C2—C3—C4—C5	−0.7 (5)	C11—C12—C13—C14	−1.1 (5)
C3—C4—C5—C6	0.3 (5)	C12—C13—C14—C15	0.4 (5)
C3—C4—C5—N1	−178.9 (3)	C12—C13—C14—N4	179.9 (3)
C7—N1—C5—C6	−166.4 (3)	C16—N4—C14—C13	17.2 (5)
N2—N1—C5—C6	12.9 (5)	N5—N4—C14—C13	−162.1 (3)
C7—N1—C5—C4	12.8 (5)	C16—N4—C14—C15	−163.2 (3)
N2—N1—C5—C4	−167.9 (3)	N5—N4—C14—C15	17.4 (5)
C4—C5—C6—C1	0.3 (5)	C13—C14—C15—C10	0.1 (5)
N1—C5—C6—C1	179.5 (3)	N4—C14—C15—C10	−179.4 (3)
C2—C1—C6—C5	−0.5 (5)	C11—C10—C15—C14	0.0 (5)
Cl1—C1—C6—C5	−179.4 (3)	Cl2—C10—C15—C14	−179.4 (3)
N2—N1—C7—C8	−0.4 (4)	N5—N4—C16—C17	0.1 (4)
C5—N1—C7—C8	179.0 (3)	C14—N4—C16—C17	−179.3 (3)
N1—C7—C8—N3	0.2 (4)	N5—N6—C17—C16	−0.3 (4)
N1—C7—C8—C9	−179.4 (3)	N5—N6—C17—C18	−179.8 (3)
N2—N3—C8—C7	0.1 (4)	N4—C16—C17—N6	0.1 (4)
N2—N3—C8—C9	179.7 (3)	N4—C16—C17—C18	179.6 (4)
C7—C8—C9—O1	−98.3 (5)	N6—C17—C18—O2	−60.3 (5)
N3—C8—C9—O1	82.2 (5)	C16—C17—C18—O2	120.2 (5)

### Hydrogen-bond geometry (Å, °)

**Table:**

*D*—H···*A*	*D*—H	H···*A*	*D*···*A*	*D*—H···*A*
O1—H1o···O2^i^	0.84 (4)	1.82 (4)	2.651 (5)	170 (5)
O2—H2o···O1w	0.84 (6)	1.80 (5)	2.641 (5)	174 (7)
O1w—H1w···N3	0.84 (4)	2.00 (4)	2.837 (5)	172 (4)
O1w—H2w···O1^ii^	0.84 (4)	1.95 (5)	2.663 (5)	142 (4)
C16—H16···O1w^iii^	0.95	2.45	3.383 (5)	166
C7—H7···N6^iv^	0.95	2.28	3.197 (5)	161

Symmetry codes: (i) −*x*+2, −*y*+1, −*z*; (ii) *x*+1, *y*, *z*; (iii) *x*−1, *y*, *z*; (iv) *x*−1, *y*+1, *z*.

## References

[bb1] Boechat, N., Ferreira, V. F., Ferreira, S. B., Ferreira, M. de L. G., da Silva, F. de C., Bastos, M. M., Costa, M. dos S., Lourenço, M. C. S., Pinto, A. C., Krettli, A. U., Aguiar, A. C., Teixeira, B. M., da Silva, N. V., Martins, P. R. C., Bezerra, F. A. F. M., Camilo, A. L. S., da Silva, G. P. & Costa, C. C. P. (2011). *J. Med. Chem.* **54**, 5988–5999. 10.1021/jm2003624 21776985

[bb2] Boechat, N., Ferreira, M. de L. G., Maria Bastos, M. M., Camilo, A. L. S., Wardell, S. M. S. V., Wardell, J. L. & Tiekink, E. R. T. (2010). *J. Chem. Crystallogr.* **40**, 1137–1141.

[bb3] Brandenburg, K. (2006). *DIAMOND* Crystal Impact GbR, Bonn, Germany.

[bb4] Costa, M. S., Boechat, N., Ferreira, V. F., Wardell, S. M. S. V. & Skakle, J. M. S. (2006*a*). *Acta Cryst.* E**62**, o1925–o1927.

[bb5] Costa, M. S., Boechat, N., Rangel, E. A., Lourenço, M. C. S., Junior, I. N., Castro, H. C., de Souza, A. M. T., da Silva, F. C., Wardell, S. M. S. V., Rodrigues, C. R. & Ferreira, V. F. (2006*b*). *Bioorg. Med. Chem.* **14**, 8644–8653. 10.1016/j.bmc.2006.08.019 16949290

[bb6] Farrugia, L. J. (1997). *J. Appl. Cryst.* **30**, 565.

[bb7] Ferreira, S. B., Costa, M. S., Boechat, N., Bezerra, R. J. S., Genestra, M. S., Canto-Cavalheiro, M. M., Kover, W. B., Vitor, F. & Ferreira, V. F. (2007). *Eur. J. Med. Chem.* **42**, 1388–1395. 10.1016/j.ejmech.2007.02.020 17445951

[bb8] Gans, J. & Shalloway, D. (2001). *J. Mol. Graph. Model.* **19**, 557–559. 10.1016/s1093-3263(01)00090-0 11552684

[bb9] Hooft, R. W. W. (1998). *COLLECT* Nonius BV, Delft, The Netherlands.

[bb10] Jordão, A. K., Afonso, P. P., Ferreira, V. F., de Souza, M. C. B. V., Almeida, M. C. B., Beltrame, C. O., Paiva, D. P., Wardell, S. M. S. V. J. L., Tiekink, E. R. T., Damaso, C. R., Anna, C. & Cunha, A. C. (2009). *Eur. J. Med. Chem.* **44**, 3777–3783. 10.1016/j.ejmech.2009.04.046 19481841

[bb11] Otwinowski, Z. & Minor, W. (1997). *Methods in Enzymology*, Vol. 276, *Macromolecular Crystallography*, Part A, edited by C. W. Carter Jr & R. M. Sweet, pp. 307–326. New York: Academic Press.

[bb12] Sheldrick, G. M. (2007). *SADABS* Bruker AXS Inc., Madison, Wisconsin, USA.

[bb13] Sheldrick, G. M. (2008). *Acta Cryst.* A**64**, 112–122. 10.1107/S0108767307043930 18156677

[bb14] Spek, A. L. (2009). *Acta Cryst.* D**65**, 148–155. 10.1107/S090744490804362X PMC2631630 19171970

[bb15] Westrip, S. P. (2010). *J. Appl. Cryst.* **43**, 920–925.

